# 76938 Does Decreased Adipose Tissue Eosinophil Content Impair Adipocyte Biology in Human Subjects with Obesity and Insulin Resistance?

**DOI:** 10.1017/cts.2021.633

**Published:** 2021-03-31

**Authors:** Eleanna De Filippis, James Hernandez, Ting Li, Elizabeth A. Jacobsen

**Affiliations:** Mayo Clinic Arizona

## Abstract

ABSTRACT IMPACT: Extrapolating from mouse data we explored eosinophil content in human adipose tissue and its effect on adipocyte biology potentially leading to the discovery of novel therapeutic targets for treatment of obesity and insulin resistance. OBJECTIVES/GOALS: The interaction between immune cells and adipose tissue (AT) in obesity has not been fully elicited. Mouse models of diet-induced-obesity show AT resident eosinophils (EOS) help preserve insulin sensitivity (IS). As data in human obesity are lacking, here we explored AT-EOS content and their role in AT metabolism in subjects with and without obesity. METHODS/STUDY POPULATION: We recruited lean (L) subjects and patients with obesity (Ob) to undergo abdominal subcutaneous AT biopsy and evaluation of insulin resistance (IR) by determination of HOMA-IR. Circulating EOS were isolated from all participants under fasting conditions and exposed to high glucose (HG) or high lipids (HL) for 4 hrs. AT EOS number was assessed via FACS analysis. Circulating EOS and AT mRNA was assessed by qPCR for multiple genes involved in inflammation and cell migration. To evaluate the effect of EOS on primary human adipocytes, in vitro cultures were exposed for 4 days to either interleukin-4 (IL-4), interleukin-13 (IL-13) or to human EOS. Adipocytes mRNA levels were evaluated for genes involved in adipogenesis and lipid metabolism. RESULTS/ANTICIPATED RESULTS: 16 lean, IS subjects (BMI 22.5+ 0.4kg/m2) and 22 age-matched IR patients with obesity (BMI: 38.9 + 1.0kg/m2) participated. We observed a ratio of 2:1 in AT EOS content of L vs Ob subjects (P<0.03). To assess the reduced AT-EOS content in obesity, we evaluated expression of Chemokine-C receptor 3 (CCR3) in circulating EOS. We show decreased CCR3 mRNA levels in Ob vs L subjects (P=0.006). We expect HL in vitro experiments on peripheral EOS of L subjects to affect CCR3 mRNA levels. In AT of Ob subjects, we found a significant decreased expression of Eotaxin 2, the main EOS chemokine binding CCR3 expressed on EOS. Preliminary data from in vitro primary adipocytes culture suggest for IL-4 and IL-13 to increase mRNA level of Peroxisome Proliferator Activated Receptor Gamma (PPARG), the master regulator of adipogenesis. DISCUSSION/SIGNIFICANCE OF FINDINGS: Comparable to animal studies, we found a decrease of AT-EOS content in patients with obesity. Alterations in CCR3/Eotaxin 2 signaling may be involved. IL-4 &IL-13 are secreted predominantly by EOS and appear to directly regulate gene expression in human adipocytes. These data represent the first evidence for a novel role of EOS in human AT biology.

